# Downstaging Therapies for Patients with Hepatocellular Carcinoma Awaiting Liver Transplantation: A Systematic Review and Meta-Analysis on Intention-to-Treat Outcomes

**DOI:** 10.3390/cancers14205102

**Published:** 2022-10-18

**Authors:** Marcello Di Martino, Alessandro Vitale, Daniele Ferraro, Marilisa Maniscalco, Donatella Pisaniello, Giuseppe Arenga, Federica Falaschi, Alfonso Terrone, Alessandro Iacomino, Alfonso Galeota Lanza, Ciro Esposito, Umberto Cillo, Giovanni Vennarecci

**Affiliations:** 1 Department of Hepatobiliary and Liver Transplantation Surgery, A.O.R.N. Cardarelli, 80128 Napoli, Italy; 2 Department of Surgical, Oncological and Gastroenterological Sciences, Padova University, 35121 Padova, Italy; 3 Division of Haepatology, A.O.R.N. Cardarelli, 80128 Napoli, Italy; 4 Liver Intesive Care Unit, Department of Hepatobiliary and Liver Transplantation Surgery, A.O.R.N. Cardarelli, 80128 Napoli, Italy

**Keywords:** hepatocellular carcinoma, cirrhosis, liver transplantation, downstaging, locoregional therapy, TACE, ablation, radiofrequency ablation, microwave ablation

## Abstract

**Simple Summary:**

Downstaging therapies provides a viable alternative approach for expanding the MC limits and selecting a subgroup of patients whose LT candidacy would otherwise be disregarded. However, data on downstaging are still controversial due to a variety of reasons, such as differences in LRT, the wide variability in waiting time before LT and, particularly, the lack of intention-to-treat (ITT) analyses. This article is a systematic review and intends to synthesise the existing evidence about the effectiveness of downstaging therapies, aiming to: (a) assess outcomes from ITT analysis of patients with liver cirrhosis and HCC beyond the listing criteria and selected for downstaging protocol, in comparison with HCC within the listing criteria; (b) evaluate outcomes of patients with liver cirrhosis HCC beyond the listing criteria successfully downstaged and transplanted versus those not transplanted.

**Abstract:**

Background: Locoregional therapies (LRTs) are commonly used to increase the number of potential candidates for liver transplantation (LT). The aim of this paper is to assess the outcomes of LRTs prior to LT in patients with hepatocellular carcinoma (HCC) beyond the listing criteria. Methods: In accordance with the PRISMA guidelines, we searched the Medline and Web of Science databases for reports published before May 2021. We included papers assessing adult patients with HCC considered for LT and reporting intention-to-treat (ITT) survival outcomes. Two reviewers independently identified and extracted the data and evaluated the papers. Outcomes analysed were drop-out rate; time on the waiting list; and 1, 3 and 5 year survival after LT and based on an ITT analysis. Results: The literature search yielded 3,106 records, of which 11 papers (1874 patients) met the inclusion criteria. Patients with HCC beyond the listing criteria and successfully downstaged presented a higher drop-out rate (OR 2.05, 95% CI 1.45–2.88, *p* < 0.001) and a longer time from the initial assessment to LT than those with HCC within the listing criteria (MD 1.93, 95% CI 0.91–2.94, *p* < 0.001). The 1, 3 and 5 year survival post-LT and based on an ITT analysis did not show significant differences between the two groups. Patients with HCC beyond the listing criteria, successfully downstaged and then transplanted, presented longer 3 year (OR 3.77, 95% CI 1.26–11.32, *p* = 0.02) and 5 year overall survival (OS) (OR 3.08, 95% CI 1.15–8.23, *p* = 0.02) in comparison with those that were not submitted to LT. Conclusions: Patients with HCC beyond the listing criteria undergoing downstaging presented a higher drop-out rate in comparison with those with HCC within the listing criteria. However, the two groups did not present significant differences in 1, 3 and 5 year survival rates based on an ITT analysis. Patients with HCC beyond the listing, when successfully downstaged and transplanted, presented longer 3 and 5-year OS in comparison with those who were not transplanted.

## 1. Introduction

Hepatocellular carcinoma (HCC) is the most common liver cancer with rising incidence over the past two decades [1]. HCC in cirrhotic patients is a leading indication for liver transplantation (LT), as it can remove the tumour and treat the liver cirrhosis [2]. Success rates of LT as a curative treatment are attributed to selective listing criteria based on morphological and biological criteria. The Milan Criteria (MC), proposed by Mazzaferro et al. [3] in 1996, have remained the benchmark for the selection of candidates for LT, being adopted by both the European Association for the Study of the Liver (EASL) [4] and the American Association for the Study of Liver Diseases (AASLD) [5] guidelines. However, the MC precludes access to LT of some patients with a potentially good outcome, and many groups have investigated how these criteria could be expanded without affecting patient survival and tumour recurrence [6,7,8,9,10,11]. Locoregional therapies (LRTs) are commonly used to increase the number of potential candidates for LT. 

Patients with liver cirrhosis and HCC initially not fulfilling the MC should not be disregarded for LT. Several reports have been published on favourable outcomes of HCC beyond the MC being successfully downstaged and then transplanted [12,13,14]. The term *downstaging therapy* refers to the process of applying LRTs to tumours currently outside the MC, with the aim of reducing tumour burden and selecting appropriate candidates for LT. Downstaging provides a viable alternative approach for expanding the MC’s limits and selecting a subgroup of patients whose LT candidacy would otherwise be disregarded. However, data on downstaging are still controversial for a variety of reasons, such as differences in LRT, the wide variability in waiting time before LT and, particularly, the lack of intention-to-treat (ITT) analyses [15,16,17]. This type of analysis assesses the results of the investigation based on the initial treatment assignment (i.e., administration of LRT) and not on the treatment eventually received (transplantation). Survival analyses from transplantation rather than ITT are prone to attrition bias and to an overestimation of the survival benefit of LRT in combination with LT for patients with HCC beyond the listing criteria. Although the effectiveness of LRT in decreasing drop-out has been documented [17] and the survival benefit has repeatedly been suggested [13,14], available data on oncological benefit based on ITT outcomes are still scarce. 

This review intends to synthesise the existing evidence about the effectiveness of downstaging therapies, aiming to (a) assess outcomes from ITT analysis of patients with liver cirrhosis and HCC beyond the listing criteria and selected for downstaging protocols, in comparison with HCC within the listing criteria; (b) evaluate outcomes of patients with liver cirrhosis and HCC beyond the listing criteria successfully downstaged and transplanted versus those not transplanted.

## 2. Materials and Methods

### 2.1. Eligibility Criteria

In order to assess the outcomes of downstaging therapies for patients with liver cirrhosis and HCC awaiting LT, two key questions were developed (Table 1). The review included studies that enrolled adults with cirrhosis awaiting LT and treated with downstaging therapies before LT and reporting outcomes based on ITT analysis. 

### 2.2. Search Strategy 

The search was undertaken according to the PRISMA guidelines [18]. Two researchers systematically searched Medline, EMBASE and the Cochrane Library for reports published before the 16th of May 2021, not limited to the English language, using a combined text and MeSH search strategy. The search terms for the literature review were divided into two groups. The first group contained the keywords downstaging, bridging, catheter ablation, chemoembolization, TACE, transarterial radioembolization, TARE, radiosurgery, radiofrequency ablation, RFA, microwave ablation, MWA, embolization, ethanol injection, PEI, high-intensity focused ultrasound ablation, high intensity focused ultrasound, HIFU, stereotactic body radiation therapy, stereotactic radiation, SBRT and radiotherapy. The second group contained the keywords hepatocellular carcinoma, HCC and liver cancer. The search terms were structured by combining one word from each group in such a way that all possible combinations were employed. References from relevant papers were also included in order to constitute the initial pool of articles.

### 2.3. Study Selection

We included full-text published studies that met the following criteria: (a) participants aged 18 years or older; (b) prospective, retrospective or randomised controlled design; (c) patients with liver cirrhosis and HCC considered for LRT and/or LT; (d) outcomes from ITT analysis reported. Survival outcomes were extrapolated from Kaplan-Meier survival curves when they were not reported. Experimental studies on animal models, case reports, short case series with fewer than 10 patients, reviews, editorials and comments were also excluded. When duplicate reports from the same study were identified, only the most recent publication or the one with the longest follow-up period was included. The full text of each article was assessed if it could not be excluded by the initial review.

### 2.4. Data Extraction

Two researchers (MDM and MM) assessed the abstracts of the selected studies to determine their eligibility. Full articles were selected for further assessment. Treatment options included the following: (a) patients with HCC beyond the listing criteria undergoing downstaging therapies before LT; (b) patients with HCC within the listing criteria treated and considered for LT; (c) patients with HCC beyond the listing criteria undergoing downstaging but not considered for LT. The extracted data included country of study; design; number of participants included; age; listing criteria; downstaging selection criteria; drop-out rate; time from listing to transplant; perioperative mortality and morbidities for patients undergoing LT; post-LT recurrence; 1, 3 and 5 year survival from LT; and 1, 3 and 5 year survival based on ITT analysis. Disagreements over data extraction were resolved by consensus between the two authors. 

### 2.5. Evaluation of Studies and Statistical Analysis

Two researchers (MDM and MM) independently evaluated the included studies for quality assessment according to either the Newcastle–Ottawa Scale [19], in the case of observational studies, or the Cochrane Risk of Bias Tool, in the case of experimental studies (Figure S1) [20]. The data were analysed using the statistical software Review Manager 5.4 and presented as medians and proportions along with a corresponding minimum–maximum range. Differences in dichotomous variables were calculated using an odds ratio (OR) and respective 95% confidence intervals (CI); for continuous variables, the mean difference (MD) was calculated with a 95% CI. A random-effects model was used to take into account the heterogeneity of the estimates. Values were considered statistically significant when p was less than 0.05. The overlapping of CI was used to visually assess the heterogeneity. Heterogeneity was statistically explored with the chi-square test, with significance set to a *p*-value of 0.10, and the quantity of heterogeneity was measured with the I^2^ statistic. The quality of evidence was estimated using the GRADE methodology, which takes into account the risk of bias, inconsistency (heterogeneity), directness of evidence, imprecision and publication bias [21]. 

## 3. Results

### 3.1. Literature Review

**Figure 1:**
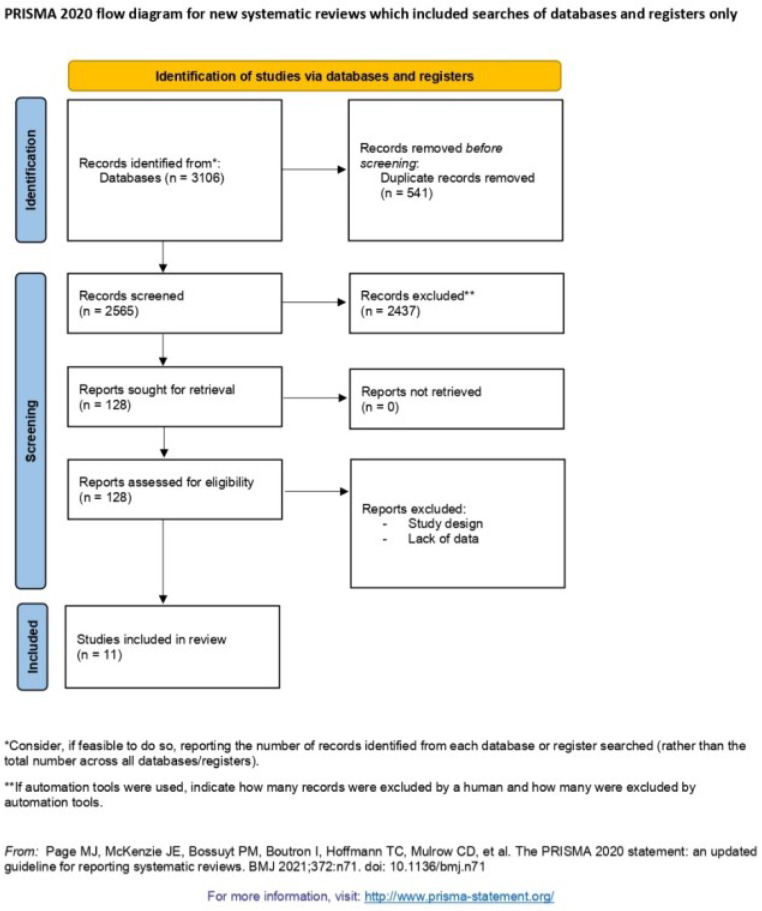
Flow chart of included studies.

The literature search yielded 3106 records, 2,978 of which were excluded due to study characteristics or methodology. The full-text articles of 128 papers were assessed for eligibility. Finally, 11 papers [15,16,22,23,24,25,26,27,28,29,30] (1874 patients) were ultimately included in the analysis (Figure 1). Eight manuscripts [15,16,22,23,24,25,26,27] (1700 patients) compared outcomes of patients with HCC outside the listing criteria submitted to downstaging in comparison with those initially within the listing criteria. Three [28,29,30] (174 patients) assessed the results of patients outside the listing criteria, downstaged and submitted to LT versus those that were downstaged but not transplanted. The reasons for exclusion were methodological issues and lack of outcomes from ITT analysis.

### 3.2. Study and Patient Characteristics

Only two randomised clinical trials were found [24,30], but none of the included papers was regarded as having a high risk of bias (Supplementary Table S1 and Figure 1). Mean age ranged from 44 to 58 years; other patient and disease characteristics in the included studies are shown in Table 2 and Table 3. LRT included transarterial embolisation (TAE), transarterial chemoembolisation (TACE), transarterial radioembolisation (TARE), radiotherapy and a sequential combination of these strategies. 

### 3.3. Patient Selection for Downstaging Therapies

When the manuscripts evaluating downstaging strategies were assessed, two were based on the University of California, San Francisco (UCSF), downstaging protocol [27,29] and two on the Bologna downstaging protocol [16,26]. Both presented precise criteria for patients’ eligibility in terms of size and number of HCC nodules. The other seven [15,22,23,24,25,28,30] did not report a downstaging protocol and did not specify eligibility criteria for downstaging strategies. However, patients responding to downstaging were finally listed for LT only if HCC nodules were within the MC (Table 2 and Table 3). 

### 3.4. Should Patients with HCC Initially beyond the Listing Criteria Be Transplanted following Successful Downstaging?

#### 3.4.1. Question 1: Comparison Based on ITT Analysis of Patients Initially beyond the Listing Criteria vs. Those within the Listing Criteria (Table 4)

(a) Waiting List Drop-out and Interval on the Waiting List

Seven studies [15,16,22,23,25,26,27] enrolled 1537 patients and reported on drop-out for any cause, four [15,23,25,26] (550 patients) reported on drop-out due to tumour progression and two [15,25] (273 patients) reported on drop-out due to tumour liver deterioration.

**Figure 2:**
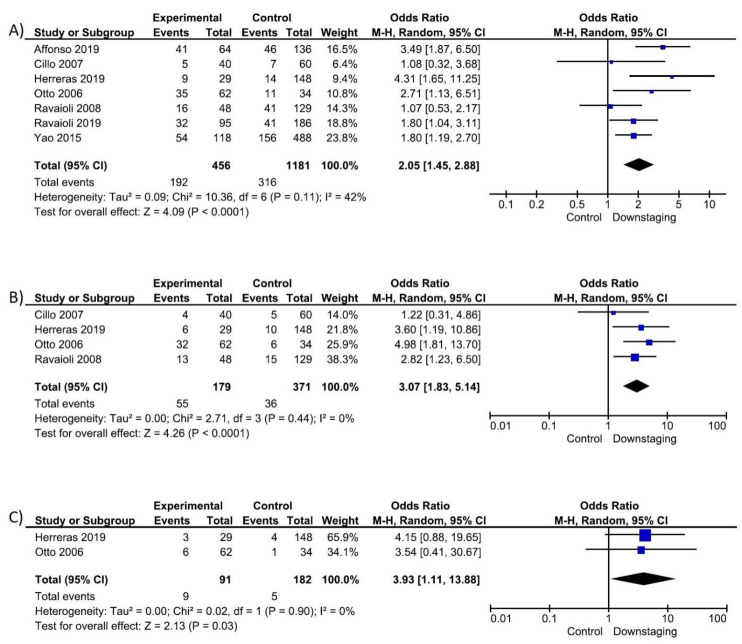
Question 1: Forrest plot on drop-out rate: (**A**) Drop-out due to all causes; (**B**) Drop-out due to tumour progression; (**C**) Drop-out due to liver deterioration.

Successful downstaging rate ranged from 87.5% [23] to 35.9% [22]. Two studies [22,25] reported a successful downstaging rate <50% after downstaging, five between 50% and 70% [15,16,24,26,27] and one [23] >70%. Patients beyond the listing criteria showed a higher total drop-out rate (OR 2.05, 95% CI 1.45–2.88, *p* < 0.001), drop-out due to tumour progression (OR 3.07, 95% CI 1.83–5.14, *p* < 0.001) and drop-out due to liver deterioration (OR 3.93, 95% CI 1.11–13.88, *p* = 0.030) (Figure 2).

**Figure 3:**
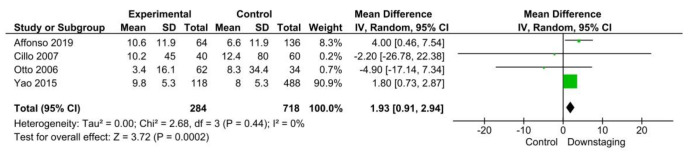
Question 1: Forrest plot on time to liver transplantation.

Additionally, four studies [22,23,25,27] (1002 patients) demonstrated that patients beyond the listing criteria presented a longer time from the first assessment to LT than those within the listing criteria (MD 1.93, 95% CI 0.91–2.94, *p* < 0.001) (Figure 3).

(b) Post-LT Survival Outcomes

**Figure 4:**
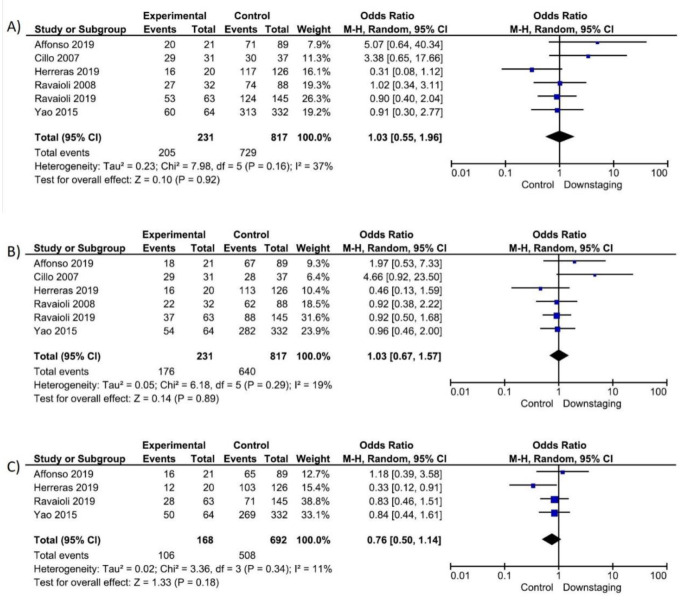
Question 1: Forrest plot post-LT survival: (**A**) 1y-overall survival; (**B**) 3y-overall survival; (**C**) 5y-overall survival.

Six studies [15,16,22,23,26,27] (1048 patients) reported on post-LT 1 and 3 year survival while four [15,16,22,27] (860 patients) reported on 5 year survival. No differences in post-LT 1, 3 and 5 year survival between the two groups were observed (Figure 4).

(c) Survival Outcomes Based on ITT Analysis

**Figure 5:**
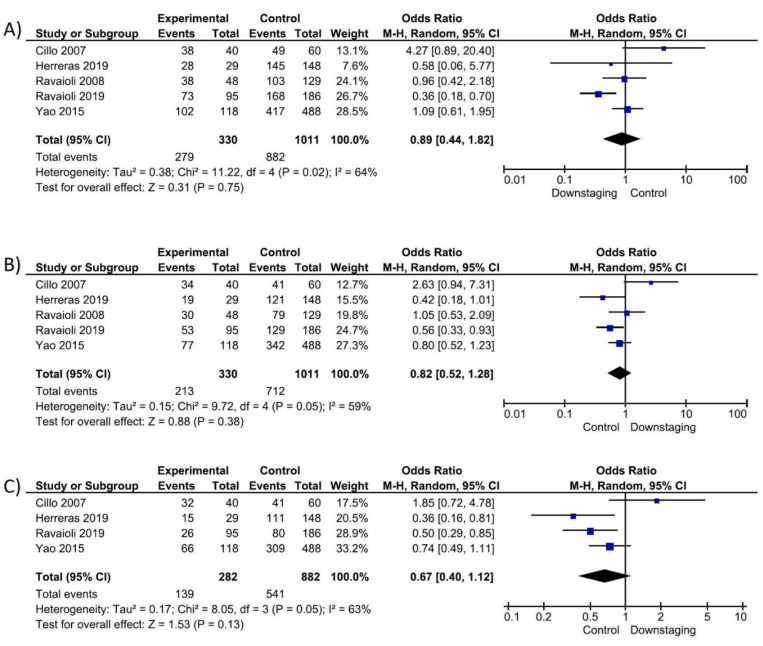
Question 1: Forrest plot of patients assessed for LT with survival based on ITT analysis: (**A**) 1y-overall survival; (**B**) 3y-overall survival; (**C**) 5y-overall survival.

Five studies [15,16,23,26,27] (1341 patients) reported on survival outcomes based on an ITT analysis, assessing 1 and 3 year survival, while four [15,16,23,27] (1164 patients) reported on 5 year survival. No differences in 1, 3 or 5 year survival outcomes based on an ITT analysis between the two groups were observed (Figure 5).

(d) Sensitivity Analysis

Three papers [16,26,27] (1064 patients) recruited patients for downstaging based on pre-specified downstaging protocols with well-defined eligibility criteria for downstaging therapies. Conversely, five studies [15,22,23,24,25] (636 patients) did not recruit patients based on a downstaging protocol; they included patients beyond the listing criteria without specifying a clear limit in terms of tumour burden. The sensitivity analysis did not show clinically relevant differences between the two subgroups. 

#### 3.4.2. Question 2: Comparison of Patients Initially beyond the Listing Criteria, Downstaged and Transplanted versus Those Not Transplanted (Table 5)

### 3.5. Survival Outcomes Based on ITT Analysis

**Figure 6:**
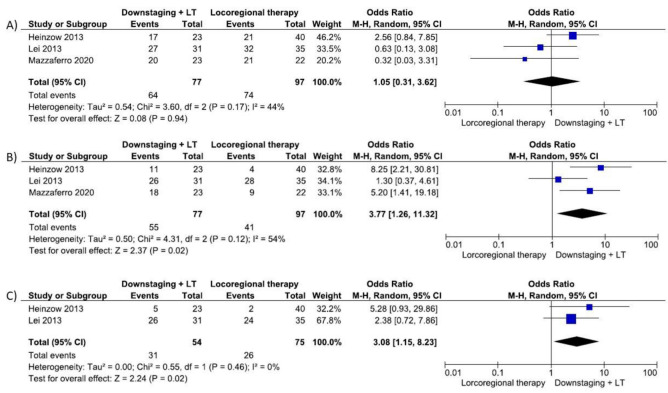
Question 2: Forrest plot post-downstaging survival based on ITT analysis: (**A**) 1y-overall survival; (**B**) 3y-overall survival; (**C**) 5y-overall survival. Bold: *p* values < 0.05.

Three studies [28,29,30] (174 patients) reported on survival outcomes based on an ITT analysis, assessing 1 and 3 year survival, while two [28,29] (129 patients) reported on 5 year survival. Patients with HCC successfully downstaged and submitted to LT presented longer 3 year (OR 3.77 95% CI 1.26–11.32, *p* = 0.02) and 5 year OS (OR 3.08, 95% CI 1.15–8.23, *p* = 0.02) in comparison with those that were not submitted to LT (Figure 6).

## 4. Discussion

Over the last few decades, several studies have postulated the benefit of LRT in patients with liver cirrhosis and HCC before LT [2,4,5,14]. However, current recommendations are mostly based on retrospective non-comparative studies with small sample sizes, short follow-up durations and reporting on post-LT outcomes. To our knowledge, this is the first meta-analysis assessing outcomes of LRT before LT according to an ITT analysis. The present analysis demonstrated that patients with HCC beyond the listing criteria submitted to a successful downstaging presented a higher drop-out rate in comparison with those with HCC initially within the listing criteria. However, the two groups did not present significant differences in survival outcomes according to an ITT analysis. This first result is extremely important when the survival benefit, which assesses the post-LT outcome, is considered the main allocation principle [31], because it showed that downstaged HCC patients can reach the same ITT survival as HCC patients transplanted within conventional criteria.

A second important result of this study, however, is that patients with HCC beyond the listing criteria when successfully downstaged and transplanted presented longer 3 and 5 year OS in comparison with those that were not transplanted. This second result is extremely important when the transplant benefit allocation principle (assessing the survival of comparable candidates with and without a transplant) is considered [32]. This result means that LT for patients with intermediate-stage HCC retains a great transplant survival benefit, probably larger than that for early-stage HCC [33,34,35]. Additionally, these findings emphasise the concept of therapeutic hierarchy [36], suggesting that patient treatment should be dictated by the efficacy of each therapy with independence from the tumour stage. In the near future, the diffusion of new systemic drugs [37,38] could increase even further the potential of downstaging therapies, increasing the number of patients with intermediate HCC likely to benefit from LT and changing the current principles of therapeutic hierarchy. 

There is still a lack of consensus on the optimal approach to patients with liver cirrhosis and HCC beyond the listing criteria. The latest EASL [4] and AASLD [5] guidelines recommend that patients beyond the MC should be considered for LT after a successful downstaging to within the MC. Successful downstaging allows time to assess the tumour response, gauge the biological behaviour and select those patients at lower risk of tumour progression [17]. Duvoux et al. [9] demonstrated that patients moving from high to low risk of recurrence, according to the AFP model, after downstaging, had the same risk of recurrence as those initially included in the low-risk category. However, there are conflicting opinions on the optimal downstaging protocols, assessment of response to downstaging and criteria for downstaging eligibility, with no universally accepted downstaging protocol [39,40,41]. In 2017, the United Network for Organ Sharing (UNOS) adopted the University of California, San Francisco (UCSF)/Region 5 downstaging protocol (UNOS-DS), the outcomes of which have recently been assessed by Mehta et al. [13]. Based on a national registry, the authors compared patients within the UNOS-DS criteria (n = 422): the ‘all-comers’ with initial tumour burden beyond UNOS-DS criteria (n = 121) and patients initially within the MC not submitted to downstaging (n = 3,276). They demonstrated a similar 3 year post-LT survival among patients with HCC always within the MC (83%) compared with those meeting UNOS-DS criteria (79%) successfully downstaged. Nevertheless, the 3 year post-LT survival in the ‘all-comers’ cohort was significantly lower, at 71%, in comparison with the two other groups. Therefore, their results presented a word of caution towards those patients beyond the UNOS-DS downstaging criteria. However, more recently, Mazzaferro et al. [30] reported the results of the first multicentric randomised clinical trial, comparing the results of LT after successful downstaging in patients beyond the MC in comparison with continuation of conventional anticancer therapies. The 5 year OS in the LT group was 77.5%, while it was 31.2% (16.6–58.5) in the control group (HR 0.32, *p* = 0.035). These findings clearly demonstrate the benefit of successful downstaging of HCC beyond the MC compared with non-transplantation therapies. The present study reinforces the evidence supporting the benefit of LRT as a downstaging strategy, demonstrating that, despite the higher rate of drop-out, patients with HCC and cirrhosis beyond the MC should still be considered for LT after successful downstaging. 

However, the present data must be interpreted with caution due to the heterogeneity of the HCC characteristics and LRT applied, the variability in waiting time before LT and the lack of standardised criteria for downstaging eligibility. As a matter of fact, this systematic review showed that the rate of successful downstaging after LRT varies widely, from 87.5% [23] to 35.9%. Patients considered for downstaging usually present intermediate-stage HCC (stage B), according to the 2022 update of the Barcelona Clinic Liver Cancer (BCLC) classification [42], which includes a great variety of tumours considered not amenable to surgical resection. According to the principle of treatment stage migration [36], stage B HCC can be downstaged with a variety of LRTs, the most commonly used alternatives being TACE and TARE. TARE has gained increasing interest in the last few years. The LEGACY study demonstrated that this therapeutic strategy could provide clinically meaningful response rates in the treatment of unresectable, solitary HCC ≤ 8 cm [43], and this recommendation was endorsed by the 2022 update of the BCLC recommendation, which included TARE as a downstaging strategy for the first time. The sensitivity analysis aimed at assessing possible differences in patients submitted to downstaging, comparing those who were treated according to a pre-specified protocol versus those who were not. However, it did not find a significant difference between the two groups. Therefore, according to the present data, despite the heterogeneity in HCC characteristics and LRT, downstaging strategies should be strongly encouraged in patients with liver cirrhosis and HCC beyond the MC. However, prospective, multicentric, well-designed studies are necessary to identify and validate reliable downstaging protocols and clarify what subgroup of patients with HCC beyond the listing criteria will present a transplant benefit from downstaging therapies.

Another important aspect to consider is that patients with HCC beyond the listing criteria successfully downstaged and transplanted presented better long-term oncological outcomes in comparison with those who were not transplanted. The findings from the present meta-analysis support the results of the recently published XXL trial by Mazzaferro et al. [30]. They randomised patients with HCC beyond MC and successfully downstaged to either being listed for LT or continuing with a non-interventional treatment. They demonstrated that patients evaluated for LT presented a superior 5 year tumour event-free survival and OS in comparison with the control group. The present systematic review identified exclusively two other additional papers assessing this issue [28,29] and confirmed that patients with HCC beyond the listing criteria downstaged and transplanted presented improved 3 and 5 year survival in comparison with those submitted to surgical resections or non-interventional treatment after the downstaging. Therefore, it seems evident that downstaging strategies and LT should be strongly encouraged in patients with liver cirrhosis and HCC beyond the MC, as they seem to offer the best chances of survival to this group of patients. 

The reported outcomes of our review should be considered and interpreted within the context of its inherent limitations. As mentioned above, there was a significant degree of heterogeneity in the inclusion and exclusion criteria of patients with HCC for downstaging and the LRT applied. For example, the downstaging therapies consisted of various form of LRT. Additionally, downstaging based on non-interventional therapies, such as systemic drugs, was not considered. Another important aspect is that, while some studies were based on precise downstaging protocols, others did not specify on what basis patients with HCC beyond the MC were selected for downstaging therapies. It must also be considered that it was not possible to consider the effects of biological markers such as alpha-fetoprotein when assessing the outcomes of patients beyond the listing criteria submitted to LRT before LT. Additionally, the vast majority of the studies included in this research were based on a small number of subjects with a retrospective non-randomised design, which are inevitably subject to selection and attrition bias. However, to the best of our knowledge, this review represents the only systematic review and meta-analysis aiming to summarise outcomes of LRT before LT based on an ITT analysis. The study design of two specific questions assessing the role of LRT and the subgroup analysis aimed to limit possible sources of heterogeneity and bias, providing consistent evidence on this issue. 

## 5. Conclusions

This analysis demonstrated that patients with HCC beyond the listing criteria undergoing downstaging before LT presented a higher drop-out rate in comparison with those initially within the listing criteria. However, the two groups did not present significant differences in post-LT and ITT 1, 3 and 5 year survival rates. At first sight, these findings can appear not too surprising. However, this study represents the first meta-analysis assessing outcomes of downstaging strategies according to an ITT analysis. It validates the principles of the treatment strategy migration and therapeutic hierarchy [36], demonstrating that downstaging strategies should be strongly encouraged. Despite the initial burden of the disease, patients successfully downstaged and transplanted presented comparable oncological outcomes with patients with HCC initially within listing criteria. Additionally, patients with HCC beyond the listing criteria, successfully downstaged and transplanted, presented significantly longer 3 and 5 year OS in comparison with those that were not transplanted. Therefore, liver transplantation, in selected patients beyond transplant criteria, can offer a demonstrable survival benefit. 

However, the lack of a homogenous downstaging protocol in the included papers obliges us to interpret this data with caution. Prospective multicentric, well-designed clinical trials should identify and validate a reliable downstaging protocol and clarify what subgroup of patients with HCC beyond the listing criteria will benefit the most from downstaging therapies.

Given the characteristics of the included papers with the downstaging group likely to include patients with more aggressive tumour biology, further RCTs do not seem to be justified, as it is highly unlikely that they would prove the superiority of the non-interventional treatment. Future research should investigate the benefits and drawbacks of specific downstaging strategies according to tumour burden and characteristics as well as the severity of the liver cirrhosis.

## Figures and Tables

**Table 1:** Population, intervention, comparison and outcomes of the proposed questions.

Question	Study Group	Intervention	Control	Outcomes
1	Patients with liver cirrhosis and HCC within and beyond listing criteria awaiting LT	Any downstaging therapy before LT (patients beyond listing criteria)	Any downstaging therapy before LT (patients within listing criteria)	Waitlist dropout, time on the waiting list, post-LT and ITT survival (1, 3 and 5 year overall survival)
2	Patients with liver cirrhosis and HCC beyond listing criteria	Any downstaging therapy before LT	Any downstaging therapy not followed by LT	ITT survival (1, 3 and 5 year overall survival)

ITT: Intention-to-treat. LT: Liver transplantation.

**Table 2:** Characteristics of included studies on Question 1: patients initially beyond listing criteria vs. those within listing criteria.

Study ID	Country	Type of Study	Total N	Inclusion Criteria Used	Criteria for Successful Downstaging	Study Group	Details	N	Control Group	Details	N	Follow-Up (Months)
Affonso 2019	Brazil	Obs. Prosp.	200	No upper limit: HCC above MC	Patients with HCC within MC	Downstaging	TACE	64	Bridging	TACE	136	
Cillo 2007	Italy	Obs. Prosp.	100	No upper limit: HCC above MC, with no extrahepatic spread, macrovascular invasion or poor differentiation.		Downstaging	Various	40	Bridging	Various	60	21
Graziadei 2003	Austria	RCT	63	No upper limit: HCC above MC, with no extrahepatic disease or vascular invasion	Patients with HCC within MC	Downstaging	TACE	15	Bridging	TACE	48	
Herreras 2019	Spain	Obs. Retrosp.	177	No upper limit: HCC above MC, with no vascular invasion, extrahepatic disease, or alpha-fetoprotein (AFP) higher than 1000 g/dL	Patients with tumour response; patients with AFP values > 400 mg/dL after downstaging procedure were excluded.	Downstaging	Various	29	Bridging	Various	148	
Otto 2006	Germany	Obs.Retrosp.	96	No upper limit: HCC above MC	Patients with tumour response.	Downstaging	TACE	62	Bridging	TACE	34	29
Ravaioli 2008	Italy	Obs. Retrosp.	177	Patient with HCC within Bologna Downstaging Protocol	Patients with HCC within MC	Downstaging	Various	48	Bridging + Obs	Various	129	
Ravaioli 2019	Italy	Obs. Retrosp.	281	Patient with HCC within Bologna Downstaging Criteria	Patients with HCC within MC	Downstaging	Various	95	Bridging + Obs	Various	186	60
Yao 2015	USA	Obs. Retrosp.	606	Patients with HCC within UCSF Downstaging Protocol	Patients with HCC within MC/UNOS T2 Criteria	Downstaging	Various	118	Bridging	Various	488	

RCT: Randomised Clinical Trial; Obs.: observational.

**Table 3:** Characteristics of included studies on Question 2: patients initially beyond listing criteria downstaged and submitted to liver transplantation vs. those downstaged and not transplanted.

Study ID	Country	Type of Study	Total N	Inclusion Criteria Used	Criteria for Successful Downstaging	Study Group	Details	N	Control Group	Details	N	Follow-Up (Months)
Heinzow 2013	Germany	Obs. Retrosp.	63	No upper limit: HCC above MC	Patients with HCC within MC	Downstaging and LT	TACE	23	Loregional therapy	TACE	40	
Lei 2013	China	Obs. Retrosp.	66	Patients with HCC within UCSF Downstaging Protocol	Patients with HCC within MC/UNOS T2 Criteria	Downstaging and LT	Various	31	Lororegional therapy and liver resection	Various	35	43
Mazzaferro 2020	Italy	RCT	45	No upper limit: HCC above MC	Patients with HCC within MC	Downstaging and LT	Various	23	Loregional therapy	Various	22	71

RCT: Randomised Clinical Trial; Obs.: observational.

**Table 4:** Question 1, summary of evidence.

	Outcomes	Studies	Patients	OR (95% CI)	I^2^	GRADE
Entire cohort	**Drop-out due to all causes**	**7**	**1537**	**2.05 (1.45–2.88)**	**42%**	⨁⨁◯◯ Low
	**Drop-out due to tumour progression**	**4**	**550**	**3.07 (1.83–5.14)**	**0%**	⨁⨁◯◯ Low
	**Drop-out due to liver deterioration**	**2**	**273**	**3.93 (1.11–13.88)**	**0%**	⨁⨁◯◯ Low
	**Time from initial assessment to LT**	**4**	**1002**	1.93 * (0.91–2.94)	**0%**	⨁⨁◯◯ Low
	Post-LT 1y-suvival	6	1048	1.03 (0.55–1.96)	37%	⨁◯◯◯ Very low
	Post-LT 3y-suvival	6	1048	1.03 (0.67–1.57)	19%	⨁◯◯◯ Very low
	Post-LT 5y-suvival	4	860	0.76 (0.50–1.14)	11%	⨁◯◯◯ Very low
	ITT 1y-suvival	5	1341	0.89 (0.44–1.82)	64%	⨁◯◯◯ Very low
	ITT 3y-suvival	5	1341	0.82 (0.52–1.28)	11%	⨁◯◯◯ Very low
	ITT 5y-suvival	4	1164	0.67 (0.40–1.12)	11%	⨁◯◯◯ Very low
Manuscript based on a downstaging protocol	**Drop-out due to all causes**	**3**	**1064**	**1.64 (1.22–2.21)**	**42%**	⨁⨁◯◯ Low
	**Drop-out due to tumour progression**	**1**	**177**	**2.82 (1.23–6.50)**	**NA**	⨁⨁◯◯ Low
	**Time from initial assessment to LT**	**1**	**606**	1.80 * (0.73–2.87)	**NA**	⨁⨁◯◯ Low
	Post-LT 1y-suvival	3	724	0.93 (0.53–1.64)	0%	⨁◯◯◯ Very low
	Post-LT 3y-suvival	3	724	0.93 (0.62–1.41)	0%	⨁◯◯◯ Very low
	Post-LT 5y-suvival	2	418	0.83 (0.54–1.30)	0%	⨁◯◯◯ Very low
	ITT 1y-suvival	3	1064	0.72 (0.35–1.47)	69%	⨁◯◯◯ Very low
	ITT 3y-suvival	3	1064	0.75 (0.54–1.04)	15%	⨁◯◯◯ Very low
	**ITT 5y-suvival**	**2**	**887**	**0.63 (0.44–0.91)**	**21%**	⨁◯◯◯ Very low
Manuscript not based on a downstaging protocol (no strict inclusion criteria for downstaging selection)	**Drop-out due to all causes**	**4**	**573**	**2.93 (1.84–4.67)**	**14%**	⨁⨁◯◯ Low
	**Drop-out due to tumour progression**	**3**	**373**	**3.13 (1.46–6.71)**	**24%**	⨁⨁◯◯ Low
	Time from initial assessment to LT	3	396	3.00 * (−0.84–6.84)	3%	⨁⨁◯◯ Low
	Post-LT 1y-suvival	3	324	1.56 (0.23–10.65)	75%	⨁◯◯◯ Very low
	Post-LT 3y-suvival	3	324	1.51 (0.40–5.72)	64%	⨁◯◯◯ Very low
	Post-LT 5y-suvival	2	196	0.61 (0.18–2.12)	64%	⨁◯◯◯ Very low
	ITT 1y-suvival	2	277	1.88 (0.27–13.16)	50%	⨁◯◯◯ Very low
	ITT 3y-suvival	2	277	1.03 (0.17–6.24)	86%	⨁◯◯◯ Very low
	ITT 5y-suvival	2	199	0.80 (0.16–4.03)	85%	⨁◯◯◯ Very low

* MD (95% CI). NA: Not Applicable. ⨁ = positive; ◯ = negative. Bold: *p* values < 0.05.

**Table 5:** Question 2, summary of evidence.

Outcomes	Studies	Patients	OR (95% CI)	I^2^	GRADE
ITT 1y-suvival	3	154	1.05 (0.31, 3.62)	44%	⨁◯◯◯ Very low
**ITT 3y-suvival**	**3**	**154**	**3.77 (1.26, 11.32)**	**54%**	**⨁◯◯◯** **Very low**
**ITT 5y-suvival**	**2**	**129**	**3.08 (1.15, 8.23)**	**0%**	**⨁◯◯◯** **Very low**

Bold: *p* values < 0.05.

## Supplementary Materials

The following supporting information can be downloaded at: https://www.mdpi.com/article/10.3390/cancers14205102/s1, Table S1: Assessment of observational studies according to the Newcastle–Ottawa Criteria. Figure S1: Assessment of experimental studies according to the Cochrane Risk of Bias Tool.
