# Supplementary material for: Downstaging Therapies for Patients with Hepatocellular Carcinoma Awaiting Liver Transplantation: A Systematic Review and Meta-Analysis on Intention-to-Treat Outcomes
Source: Cancers (Basel). 2022 Oct 18;14(20):5102. doi: 10.3390/cancers14205102 (PMC9600776; doi:10.3390/cancers14205102)
Supplement: Supplementary file 1 [file cancers-14-05102-s001.zip › cancers-1952313-supplementary.pdf]

Table S1. Assessment of observational studies according to the Newcastle–Ottawa Criteria.

	Selection - Representativeness of exposed cohort: Truly representative of the average patients listed for LT in the community (1), Somewhat representative of the average patients listed for LT in the community (1), Selected group of users (nurses, volunteers), No description of derivation of cohort	Selection - Representativeness of non-exposed cohort: Drawn from same community as exposed cohort (1), Drawn from a different source, No description of the derivation of non-exposed cohort	Selection - Ascertainment of exposure: Secure record (surgical records) (1), Structured interview (1), Written self-report, No description	Selection - Demonstration that outcome of interest was not present at start of study: Yes (1), No	Comparability - Comparability of cohorts on the basis of design or analysis: Study controls for type of indication for LT (1), Study controls for >1 other well-established predictors of survival (etiology, stage, type of LT) (1)	Outcome - Assessment of outcome: Independent blind assessment (1), Record linkage (1), Self-report , No description	Outcome - Was follow-up long enough for outcomes to occur: Yes, follow-up at least for 60 months (in order to determine 5-year survival) (1), No, follow-up less than 60 months	Outcome - Adequacy of follow-up of cohorts: Complete follow-up (all subjects accounted for) (1), Subjects lost to follow-up unlikely to introduce bias – small number lost to follow-up (1), Follow-up rate likely to introduce bias with no description of those lost to follow-up, No statement	Total
Affonso 2019	1	1	0	1	1	0	1	1	6
Cillo 2007	1	1	1	1	1	0	1	1	7
Herreras 2019	1	1	1	1	1	0	1	1	7
Otto 2006	1	1	1	1	1	0	0	1	6
Ravaioli 2008	1	1	1	1	1	0	0	1	6
Ravaioli 2019	1	1	1	1	1	0	0	1	6

Yao 2015	1	1	1	1	1	0	1	1	7
Heinzow 2013	1	1	0	1	1	0	1	1	6
Lei 2013	1	1	0	1	1	0	1	1	6

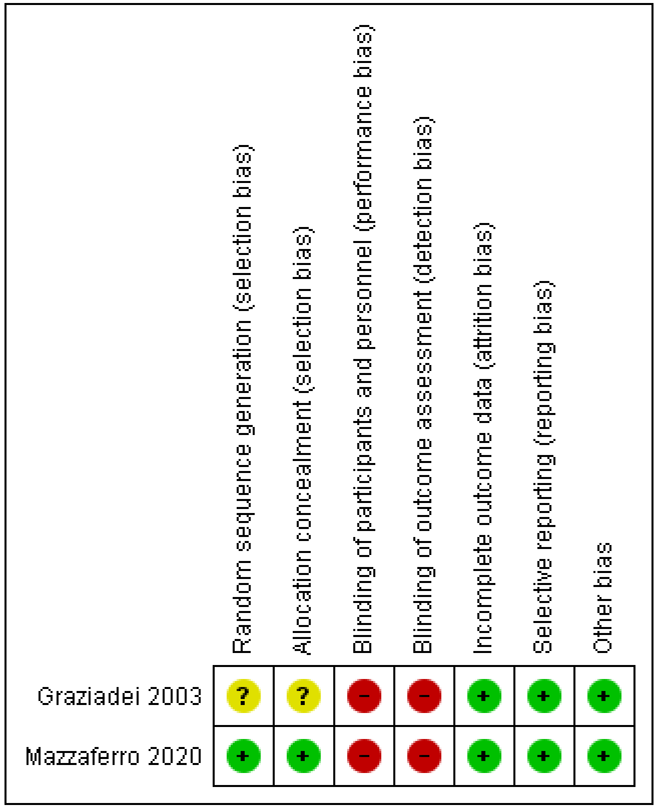

Figure S1. Assessment of experimental studies according to the Cochrane Risk of Bias Tool.
